# Complete Resolution of the Pituitary Mass Lesion and Improvement of Pituitary Function with Corticosteroid in Autoimmune Hypophysitis: A Case Report

**DOI:** 10.4314/ejhs.v31i5.20

**Published:** 2021-09

**Authors:** Kishore Kumar Behera, Ranjan Kumar Jena, Subhendu Kumar Sahoo, Uttam Kumar Soren

**Affiliations:** 1 Departments of Endocrinology and Metabolism, All India Institute of Medical Sciences, Bhubaneswar-751019 (India); 2 Department of Neurosurgery, All India Institute of Medical Sciences, Bhubaneswar-751019 (India)

**Keywords:** Autoimmune hypophysitis, pituitary mass lesion, glucocorticoid, panhypopituitarism

## Abstract

**Background:**

Autoimmune hypophysitis is the consequence of an immune-mediated inflammation of the pituitary gland, which is rare, and most frequently occurs in females during postpartum periods. It usually responds well to corticosteroid treatment with reported resolution of the pituitary mass lesion.

**Case Report:**

A 51 years male presented with a one-month history of lethargy, headache, nausea, proximal muscle weakness with intermittent flushing. He was a diabetic with metformin 500mg twice daily. No other remarkable medical history or family history of autoimmune disease. On examination, he had no neurological deficit with a normal visual field. His initial biochemical evaluation showed features of secondary hypothyroidism as evidenced by low free FT4 and suppressed TSH with normal electrolytes. The subsequent evaluation of his hormonal profile revealed panhypopituitarism. Contrast MRI of pituitary showed an enhanced homogenous mass and minimal stalk thickening with a dural tail and preserved posterior bright spot. He was managed with glucocorticoid 20 mg once daily for two weeks along with levothyroxine and testosterone replacement. After two weeks of treatment, he improved clinically. Repeat MRI imaging of the pituitary showed complete resolution of the homogenous mass.

**Conclusion:**

Although autoimmune hypophysitis is rare in males, a careful clinical history with necessary hormonal investigations is required for the suspicion about the inflammatory pituitary disorders This current case highlights glucocorticoid as the primary modality of treatment and the need for long-term follow-up with periodic clinical assessment.

## Introduction

Hypophysitis is an inflammation of the pituitary gland of acute or chronic onset. Hypophysitis can be further classified based on its cause, involvement of various parts of pituitary glands, and/or histology characteristics. Primary hypophysitis (lymphocytic or autoimmune) refers to isolated lymphocytic inflammation and infiltration of the pituitary not associated with other known causes of pituitary inflammation (1).

Anatomically hypophysitis is classified according to involvements of various parts of pituitary glands such as involvement of the anterior pituitary gland (adenohypophysitis), posterior gland, and stalk (infundibuloneurohypophystis), or entire gland (panhypophysitis) (1,3). Histologically hypophysitis can be classified into the following subtypes: lymphocytic, granulomatous, xanthomatous, and plasmocytic rarely mixed type (3).

Autoimmune hypophysitis (AH) is an uncommon inflammatory disorder, mostly encounters young females, especially in the peripartum period. It was first recognized in 1962 by Goudie and Pinkerton (1,2). Our case is a 51-year-old man with suspected AH that was reported here in detail of mode of diagnosis, treatment, and timeline of follow-up.

## Case Report

A fifty-one years old medical professional presented with a month of history of lethargy, frontal headache, proximal muscle weakness, flushing with nausea. His medical history was unremarkable other than T2DM 3month back with metformin 500mg twice daily. No personal or family history of autoimmune disease in past or the family. He was referred here for further evaluation.

He has been examined thoroughly without any focal neurological disorder. Ophthalmology examination revealed a normal visual field. Initial blood report shows secondary hypothyroid as evidenced by low free T4 and suppressed TSH with normal serum electrolytes table 1B; hence a complete hormonal test was ordered.

**Table 1:** IA – showing hormonal values at basal and 1month after hormone replacement, 1B- showing biochemistry profile blood and urine;1C- autoimmune markers

Test	August 2020	September 2020	Normal Range
**1A-Hormone Profile**			
TSH	0.065	2.136	0.34 – 5.5(µIU/ml)
Free T4	0.63	1.42	0.89 – 1.76(ng/dl)
Free T3	3.57	2.81	2.3 – 4.29(pg/ml)
Cortisol	1.35	1.48	5 – 23(µgm/dl)
LH	0.47	3.02	1.24–7.8 (mIU/ml)
FSH	0.79	7.02	1.5 -12.4(mIU/ml)
Prolactin	3.11	10.32	< 20 (ng/ml)
Testosterone	45.20	551.56	241 – 827(ng/dl)
**1B -Biochemistry Profile**		Normal range	
**Sodium**	138	137	135 – 145(mmol/l)
**Potassium**	3.70	3.8	3.5 – 5.5(mmol/l)
**TC**	150	188	< 200(mg/dl)
**TG**	610	119	< 150(mg/dl)
**HDL**	46	67	40 -60(mg/dl)
**LDL**	110	99	100 -129(mg/dl)
**FBS**	110	95	70 -110(mg/dl)
**PPBS**	210	265	70 -140(mg/dl)
**HbA1C**	7.5	7.6 (%)	
**Urea**	32	39	17.0 – 43.0(mg/dl)
**Creatinine**	1.1	1.2	0.7 – 1.3(mg/dl)
**Urine Microalbumin**		4.54	
**Urine Albumin to**		5.675	< 30(mg/gm)
**Creatinine ration**			
**1C-Autoimmune Profile**			
**ANA**	Negative		
DsDNA	Negative		
**TPO**	28.0		
**CRP**	Negative		0.6 mg/dl
**P-ANCA**	Negative		
**C-ANCA**	Negative		

**Figure 1A:**
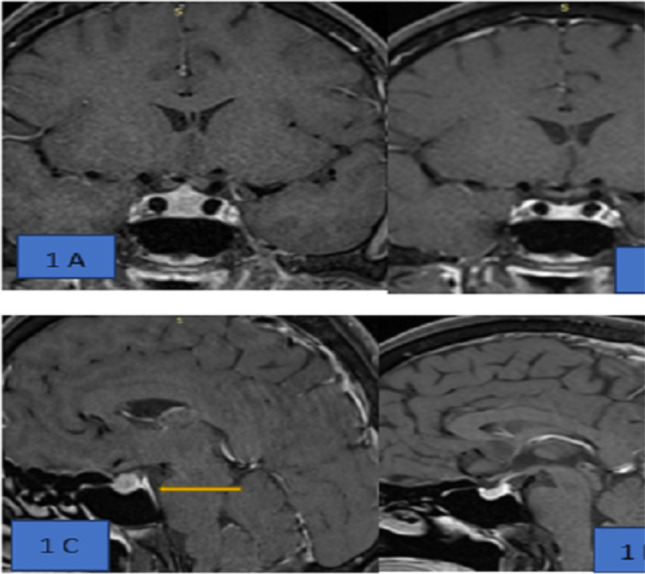
Pituitary mass lesion with stalk thickening; I-B & I-D-showing complete resolution of mass lesion with glucocorticoid therapy, 1-C- showing dural tail and posterior pituitary bright spot

His hormonal reports are depicted in table 1A; which is indicative of panhypopituitarism. An MRI with a contrast of the pituitary showed an enhanced homogenous mass lesion with minimal stalk thickening with dural tail (fig-1C) with a preserved posterior bright spot. He was neither having hypernatremia nor polyuria, so central diabetes insipidus was excluded. He was advised glucocorticoid 20 mg once daily for two weeks along with levothyroxine replacement for central hypothyroidism.

**Follow-up**: After two weeks of treatment, he improved symptomatically. A repeat MRI imaging of the pituitary showed complete resolution of the homogenous mass (Figures 1-b; 1c) with the recovery of the tropic pituitary hormone as shown in table 2. He had taken prednisolone 20 mg once daily for two weeks then gradually tapered for 4weeks, so a total of 6 weeks of steroid was advised. He was reassessed thereafter which showed recovery of the hypothalamus-pituitary-adrenal axis. There was no biochemical or clinical feature of diabetes insipidus during glucocorticoid treatment. In the literature the recurrence of hypophysitis is a quite common phenomenon, so he was told to remain under regular follow-up.

**Table 2:** Hormonal profile at baseline and following appropriate hormonal replacement

Test	At baseline	6 Weeks of steroids	2 Weeks after Stoppage steroid	Normal range
TSH	0.065	2.136		0.34 – 5.5 (µIU/ml)
Free T4	0.63	1.42		0.89 – 1.76 (ng/dl)
Free T3	3.57	2.81		2.3 – 4.2 (pg/ml)
Cortisol	1.35	1.48	5,15 *	5.0 – 23.0 (µg/dl)
LH	0.47	3.02		1.24 – 8.0 (mIU/ml)
FSH	0.79	7.02		1.5 – 12.4 (mIU/ml)
Prolactin	3.11	10.32		< 20 (ng/ml)
Testosterone	45.20	551.56	560.23	241–827 (ng/dl)
TSH	0.065	2.136		0.34–5.5 (mIU/l)
ACTH			36	30–40 (pg/ml)

## Discussion

The exact cause underlying the development of hypophysitis is inconclusive. Pituitary histopathology examination is the gold standard for diagnosis of AH but demands expertise to get the tissue from pituitary lesions. At present, with a better understanding of natural history, advance in imaging modality and characteristic MRI features the diagnosis of AH can be done with certainty in the absence of histology diagnosis. There is evidenced from the literature (4) that the diagnosis and treatment of AH based on excluding the other underlying aetiologies of hypophysitis; in addition to taking account of clinical, biochemical, and radiological criteria and response to steroid therapy [5] which potentially avoid surgical intervention with the associated risk.

Our case had an excellent response with glucocorticoid (GC) therapy with a complete resolution of mass lesion in the pituitary (fig-1B,1D) with the improvement of pituitary function(tab-1A). Due to the excellent response to the GC and the absence of other systemic inflammatory biomarkers (1C), he was diagnosed with AH.

There are three modalities of treatment for AH; medical treatment with oral GC or pulse methylprednisolone, immunosuppressive therapy for the GC resistant case, and finally surgery is reserved for those patients who require diagnostic confirmation or decompression of the optic chiasm.

A 62.5%, and 44% response to treatment rate with a pharmacological dose of prednisone equivalent to >10 mg/day, and <10 mg/day was reported respectively (3). We started 20mg of GC because the index case was a diabetic and dyslipidemia on treatment.

There is another form of intervention in AH that has been tried such as stereotactic radiosurgery and radiotherapy in a few cases who show recurrence after surgery and are resistant to glucocorticoids [3,5].

## Conclusion

Our case demonstrates the importance of clinical history and hormonal investigation for the suspicion about the inflammatory pituitary disorders of autoimmune nature which is a diagnosis of exclusion of other inflammatory disorders of the pituitary gland. The current case highlights glucocorticoid as the primary modality of treatment and the need for long-term follow-up with periodic clinical assessment; if deem necessary an MRI imaging to find out the progression of a mass lesion.
